# Social networks of oncology clinicians as a means for increasing survivorship clinic referral

**DOI:** 10.1038/s43856-022-00153-0

**Published:** 2022-07-15

**Authors:** Sarah E. Piombo, Kimberly A. Miller, David R. Freyer, Joel E. Milam, Anamara Ritt-Olson, Gino K. In, Thomas W. Valente

**Affiliations:** 1 Department of Population and Public Health Sciences, Keck School of Medicine of the University of Southern California, Los Angeles, CA USA; 2 Department of Dermatology, Keck School of Medicine of the University of Southern California, Los Angeles, CA USA; 3 Department of Medicine, Keck School of Medicine of the University of Southern California, Los Angeles, CA USA; 4 USC Norris Comprehensive Cancer Center, Los Angeles, CA USA; 5 Cancer and Blood Disease Institute, Children’s Hospital Los Angeles, Los Angeles, CA USA; 6 Department of Epidemiology, School of Population and Public Health, University of California Irvine, Irvine, CA USA; 7 Department of Health, Society, and Behavior, School of Population and Public Health, University of California Irvine, CA Irvine, USA

**Keywords:** Health services, Cancer

## Abstract

**Background:**

Specialized cancer survivorship clinics are recommended for addressing treatment-related health concerns of long-term survivors, but their relative newness in medical oncology necessitates strategies to expand services and clinic referrals. This study used social network analysis to identify personal and/or network factors associated with referral of patients to a survivorship clinic.

**Methods:**

We conducted a cross-sectional social network survey of clinical personnel at a National Cancer Institute-designated comprehensive cancer center. Participants identified colleagues with whom they consult for advice (advice network) and/or discuss patient care (discussion network). Exponential random graph models and logistic regression were used to identify key opinion leaders in the network and factors associated with referral of patients to the center’s survivorship clinic.

**Results:**

Here we show that of the respondents (n = 69), 78.0% report being aware of the survivorship clinic, yet only 30.4% had ever referred patients to it. Individuals tend to associate with others in the same occupational role (homophily). In the discussion network, holding an influential network position (betweenness centrality) is associated with patient referral to the survivorship clinic. In the advice network, several social workers are identified as opinion leaders.

**Conclusions:**

This study shows that there is strong homophily in both networks, potentially inhibiting information sharing between groups. In designing an inclusive network intervention, persons occupying influential network positions and opinion leaders (e.g., social workers in this case) are well-positioned to promote survivorship clinic referrals.

## Plain language summary

Specialized cancer survivorship clinics are an attractive but under-utilized option for monitoring long-term survivors at risk for developing serious health problems after treatment. The purpose of this survey-based social network analysis was to describe clinician interactions within a cancer center and explore opportunities for developing interventions to increase survivorship clinic referrals. We asked cancer clinicians to identify which colleagues they consult for advice about patient care and whether they were already referring survivors to the clinic. We found that clinicians in central ‘gatekeeper’ positions were more likely to refer survivors and facilitate information sharing among clinician groups. Social workers were sought most often for advice about patient care, suggesting an intervention utilizing these professionals could potentially raise awareness about survivorship clinic among colleagues and result in increased referrals.

## Abstract (web summary)

Piombo et al. analyse social networks of cancer clinicians to study referral practices to a cancer survivorship clinic at a comprehensive cancer centre. The authors identify key opinion leaders within the networks and suggest interventions to improve referrals to survivorship services.

## Introduction

Due to recent advances in diagnosis and therapy, most adults treated for cancer now achieve long-term survival with 84.6% of patients aged 19–39 years, and 72.7% of patients aged 40–64 years, living 5 years or longer^1^. Unfortunately, many survivors develop clinically significant health problems as a result of cancer treatment, resulting in physical symptoms, functional impairment, early mortality, diminished health-related quality of life, emotional distress, barriers to school and work, and financial insecurity^2^. To address these issues, specialized survivorship clinics are recommended as a means for adult cancer survivors to obtain critical services including medical monitoring and management of chronic side effects, psychosocial support, fertility assistance, and written survivorship care plans summarizing prior treatment and recommendations for improving health^3–7^.

Cancer survivorship is a relatively new discipline within adult oncology and the availability of specialized clinics for adult cancer survivors is limited. This is in contrast to pediatric oncology, where survivorship clinics are ubiquitous and their role well-established due to historically high long-term survival rates of children with cancer, their high prevalence of late effects, extensive survivorship research, and availability of long-term follow-up guidelines^8–12^. Building on this, the National Comprehensive Cancer Network and the American College of Surgeons have prioritized provision of appropriate adult-focused survivorship care^7,13,14^. Therefore, effective strategies are needed for cultivating survivorship services in adult cancer centers, including optimizing referrals to cancer survivorship clinics, which have been identified as an effective model for delivering survivorship care^6^.

However, implementation of new clinical practices and acceptance of new procedures can be a lengthy process^15^. One innovative approach to understanding and potentially impacting the dynamics of referrals to survivorship clinics is through social network analysis. Social network analysis is a scientific discipline that explores communication patterns, diffusion of ideas and innovations, and the adoption of new practices^16^. Social network analysis and constructs have been used in clinical and medical settings since the 1950s to understand the adoption of new practices among physicians^17^. Social network-based interventions have been used to promote changes in guideline compliance, prescribing practices, and implementation of evidence-based medicine among clinicians^18–21^. This methodology is critical to understanding the effects of social influence on the dissemination of new ideas and behaviors in clinical settings and can be used to implement change.

This framework provides insight into communication patterns within networks and identifies central individuals who are best able to influence others to change their behavior^22–24^. These individuals are opinion leaders who interact with many others and/or whom others consult for advice. Professional *advice networks* typically capture the relationships people seek out when they need guidance from someone whose expertize and knowledge they value on a certain topic^25^. Advice network nominations, often to colleagues of higher rank or prestige, can be used to identify *opinion leaders* who can act as agents of change in network interventions^16^. In contrast, discussion networks are often to colleagues/peers of similar rank or prestige and capture relationships with individuals to discuss their work, but not necessarily to seek advice or expertize (see Supplementary Table 1 for glossary terminology).

Social network dynamics have been shown to impact health outcomes and utilization of healthcare^26,27^. Social network analysis has been used to characterize provider collaborations in breast cancer care^28^ and initiatives for reducing cancer care disparities^29^, but not, to our knowledge, in cancer survivorship care. The aim of this study was to identify the opinion leaders in oncology clinician advice and discussion networks regarding referral of patients to a newly-established survivorship clinic at a National Cancer Institute (NCI)-designated comprehensive cancer center. We hypothesized that network members would cluster based on both clinical roles and patient referral patterns. The overall goal was to identify personal and/or network factors that were associated with survivorship clinic referrals and are relevant to development of a network intervention to increase clinic referrals. Results show evidence of role homophily in both the advice and discussion networks, potentially inhibiting the flow of communication between individuals in different clinical roles. Individuals with high betweenness centrality, who occupy bridging positions, were significantly more likely to refer patients. Additionally, social workers emerged as opinion leaders in the advice network and may be influential in promoting clinic referral.

## Methods

### Study design, setting, participants and procedures

The study comprised a cross-sectional survey of clinicians and clinical support staff who could potentially refer patients to the cancer survivorship clinic at the Norris Comprehensive Cancer Center. Started in 2017, the cancer survivorship clinic provides in-depth, post-treatment assessments for cancer survivors with the goal of improving health outcomes. Referral guidelines specify patients treated with curative intent at less than 50 years of age using cancer therapy associated with long-term toxicity.

Eligible clinicians included treating physicians (medical, surgical, and radiation oncologists), physician assistants, nurse practitioners, oncology clinic nurses, nurse navigators, social workers, and genetic counselors actively practising at the cancer institute. Eligible clinical support staff included schedulers, direct care partners, and clerical referral specialists. All clinicians and staff had regular, direct contact with patients who met cancer survivorship clinic referral guidelines and played a role in the cancer survivorship clinic referral process. Overall, 163 eligible individuals were identified from medical staff lists, department rosters, and managers and invited to participate. Recruitment was purposeful to include a representative range of cancer- and modality-specific treatment teams, departments, and disciplines.

Potential participants were invited by email to complete a confidential online survey through Qualtrics about professional social networks and patient referrals (See Supplementary Note 1). Data collection occurred from June 2018 to August 2018. Participants were given an information sheet and electronically consented to participate in the study and were compensated with a $10 gift card for survey completion. The study was approved by the University of Southern California Institutional Review Board (approval number HS-09-00673).

### Measures

#### Cancer survivorship clinic knowledge and utilization

Participants were asked: (1) if they were aware that the survivorship clinic existed (yes/no/not sure); (2) if they had referred any patients to the survivorship clinic (yes/no/not sure); (3) if they had referred any patients who met clinic guidelines in the past 12 months; and (4) to estimate how many patients they have referred to the clinic in the past 12 months.

#### Advice network

Participants were asked to name up to seven individuals at the cancer center whom they *go to for advice about any aspect of patient care for their cancer patients*. Individuals could be from any clinical role, profession, or occupation. Nominations create a connection between two individuals in a network, referred to as a network tie. Advice network nominations were used to create a directed adjacency matrix, where each directed pair of individuals *x*_*ij*_ = 1, if participant *i* nominated individual *j* as a person they work with and go to for advice about patient care.

#### Discussion network

Participants were asked to name up to seven individuals at the cancer center with whom they *discussed any aspect of patient care for their cancer patients*. Individuals could be from any occupation at the cancer center, not necessarily the same occupation as oneself. The same individuals could be nominated for both the advice and discussion networks. Discussion network nominations were used to create a directed adjacency matrix, where each directed pair of individuals *x*_*ij*_ = 1, if participant *i* nominated individual *j* as a person they discuss patient care with.

#### Network exposure

Network exposure^30^ was calculated as the proportion of individuals in one’s network that reported referring patients to the survivorship clinic (e.g., if someone nominated 6 individuals, and 3 of these nominated individuals had referred patients to the clinic, then personal network exposure would be 0.50).

### Statistical analysis

Discussion and advice networks were analyzed separately using exponential random graph models (ERGMs)^31,32^. In ERGMs, the dependent variable is the presence or absence of a tie between two people in the network (1 = present, 0 = absent). ERGM estimation and interpretation is similar to logistic regression. However, ERGMs are unique in that they control for dependencies among observations since there are multiple observations for each member of the network. Additionally, individual attribute and network structural effects are estimated in the same model. ERGMs model the probability of a tie in the observed network occurring more or less often than would be expected by chance while controlling for network density (the number of ties in the network) and network structural effects (i.e., reciprocity or mutual connections). Maximum likelihood estimates for the ERGMs were fit separately for advice and discussion networks using Markov Chain Monte Carlo in R (version 4.1.2).

Advice and discussion networks were restricted to people who completed the survey. To explore the relationships among network ties and participant attributes, an effect was added to each model matching for role at the cancer center, awareness of the survivorship clinic (Yes = 1, No = 0) and referral of patients to the survivorship clinic (Yes = 1, No = 0). Structural effects were added for tie reciprocity, and geometrically weighted edgewise shared partnership (Gwesp), where people have indirect ties throughout the network in common, and geometrically weighted outdegree distribution (Gwodegree), a measure for outdegree distribution in the network. Additional combinations of structural terms (Gwesp, Gwodegree, Gwidegree) were tested but model convergence was not reached. In addition to the exponential random graph models, multivariable logistic regression was used to analyze factors associated with referral of patients to the survivorship clinic while controlling for covariates.

### Reporting summary

Further information on research design is available in the Nature Research Reporting Summary linked to this article.

## Results

### Descriptive statistics and network characteristics

Of the 163 people invited, 69 participants provided sufficient demographic and network data to be included in the analysis, yielding a 42% response rate. Descriptive statistics of the analytic sample are presented in Table 1. The majority of the sample consisted of schedulers/other roles at the hospital (31.2%) and physicians (29.0%). “Other” roles included genetic counselors, direct care partners, and clerical referral coordinators. Physicians included medical, surgical, and radiation oncologists from various disciplines. While 78.0% of participants reported being aware of the survivorship clinic, only 30.4% had ever referred patients. Of the non-referrers, most were physicians, advanced practice providers, and nurses. The average *indegree*, or number of nominations received, was 1.6 for the advice network and 2.0 for the discussion network.

**Table 1:** Participant characteristics (*N* = 69)^a^.

	*N* (%)
Role at cancer center	
Scheduler/other^b^	22 (31.2)
Physician	20 (29.0)
Clinic nurse	16 (23.2)
Social worker	6 (8.7)
Physician assistant/nurse practitioner	5 (7.3)
Aware of survivorship clinic	
Yes	54 (78.3)
No	8 (11.6)
Don’t know	3 (4.4)
Missing	4 (5.8)
Have referred patients to the survivorship clinic	
Yes	21 (30.4)
No	39 (56.5)
Don’t know	4 (5.8)
Missing	5 (7.3)

^a^Includes only those participants who provided responses for both the advice and discussion networks.

^b^Includes genetic counselors, direct care partners, and clerical referral specialists.

### Social networks

**Fig. 1:**
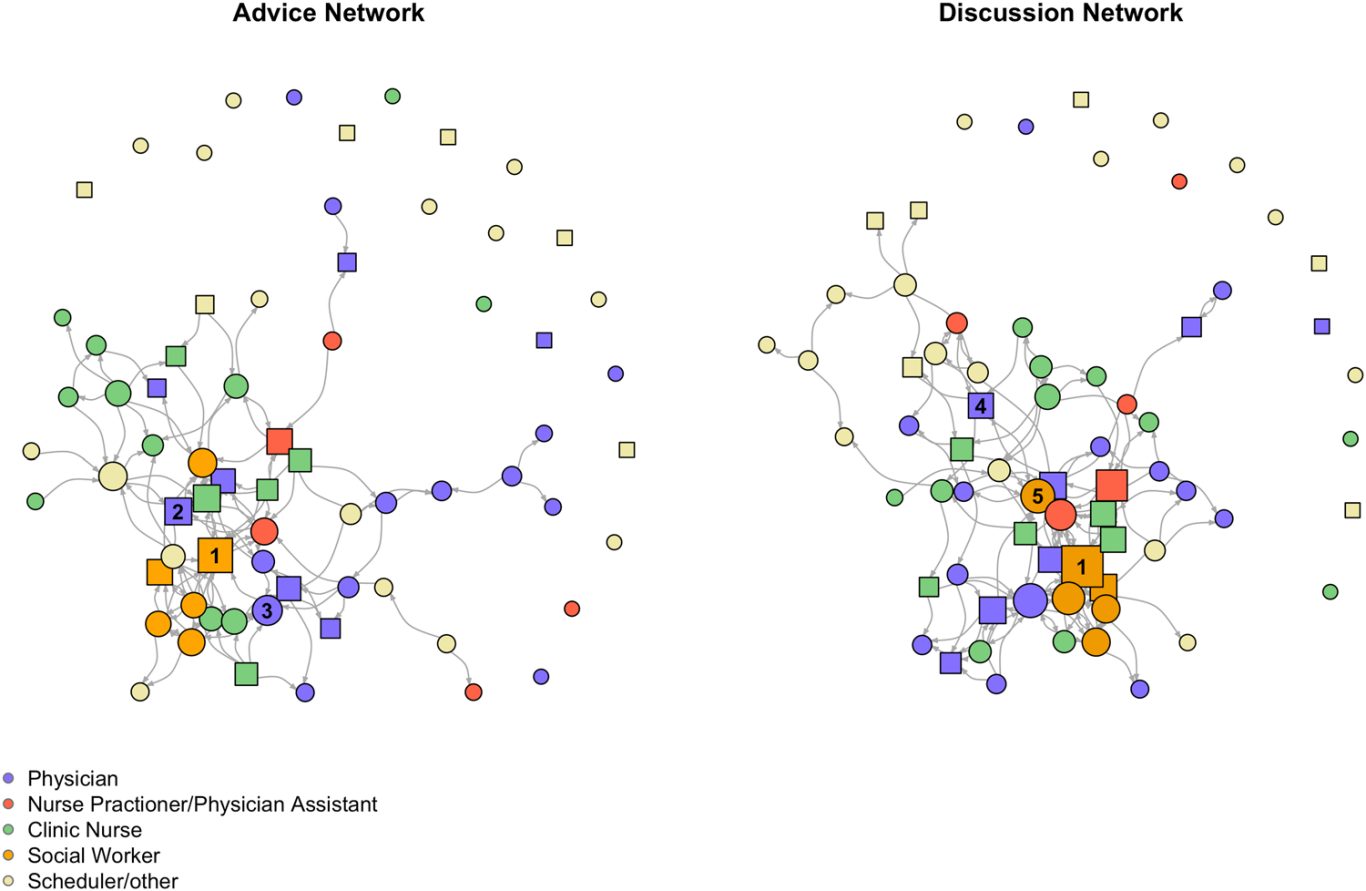
Advice and discussion networks of oncology clinicians. Nodes sized proportionate to indegree, i.e., the number of times nominated. Squares indicate people who have referred patients to the survivorship clinic, circular nodes have not previously referred patients to the clinic. The numbered nodes are the individuals with the highest betweenness centrality.

The advice and discussion network plots are shown in Fig. 1. Individuals in the network that participants reported they most frequently turned to for advice on patient care, or *opinion leaders*, were identified by indegree. The individuals with the highest indegree in the advice network were two social workers with indegrees of 11 and 7, respectively, and a medical oncologist with an indegree of 8. In the discussion network, which measures with whom individuals discuss patient care, the individual with the highest indegree was one of the same social workers with an indegree of 12, and a different social worker and medical oncologist, both with indegrees of 9.

### Exponential random graph models

The results of the ERGMs fit for the advice and discussion networks are shown in Table 2. Attribute effects show that social workers and nurse practitioners were significantly more likely to receive nominations. Participants were significantly more likely to be connected to others if they had shared indirect connections or ties (Gwesp), and/or the same occupational role at the clinic, an effect known as homophily (i.e., physicians were more likely to be connected to other physicians, and nurses to other nurses, etc.). The significant effect for geometrically weighted outdegree distribution indicates that there is an uneven distribution in the number of nominations by the respondents. As the overall network is sparse, the significant effect for geometrically weighted outdegree distribution in the number of nominations by the respondents results in a more centralized network compared to a random one.

**Table 2:** Exponential random graph models for advice and discussion networks.

	Advice		Discussion	
	Estimate (SE)	*P*-value	Estimate (SE)	*P*-value
Structural effects				
Edges	−4.97 (0.55)	<0.0001	−4.91 (0.56)	<0.0001
Mutuality	0.65 (0.47)	0.16	1.52 (0.36)	<0.0001
Gwesp	1.15 (0.18)	0.0004	1.02 (0.16)	<0.0001
Gwodegree	−1.47 (0.42)	<0.0001	−1.33 (0.41)	0.001
Attribute effects				
Physicians^a^	−0.02 (0.07)	0.79	0.01 (0.08)	0.93
Nurse practitioner/physician assistant	0.31 (0.15)	0.04	0.37 (0.13)	0.004
Social workers	0.38 (0.10)	0.0003	0.52 (0.14)	0.0002
Scheduler/other	−0.31 (0.15)	0.04	−0.26 (0.13)	0.05
Awareness of survivorship clinic	0.45 (0.45)	0.32	0.59 (0.50)	0.24
Referral to survivorship clinic	0.10 (0.10)	0.31	0.13 (0.10)	0.20
Matched				
Node match role at clinic	0.88 (0.18)	<0.0001	0.79 (0.15)	<0.0001
Node match awareness	−0.05 (0.52)	0.93	−0.42 (0.54)	0.43
Node match referrals	0.19 (0.20)	0.33	0.08 (0.17)	0.66

^a^Clinic nurses as reference group.

In the discussion network, ties were more likely to be reciprocal, and participants were more likely to be connected if they shared indirect ties (Gwesp), and the geometrically weighted outdegree distribution (Gwodegree) was also significant as in the advice network. Nurse practitioners, physician assistants, and social workers were significantly more likely to receive discussion nominations, while schedulers and other roles were significantly less likely. Similar to the advice network, role homophily was present.

### Logistic regression

Multivariable logistic regression was used to identify factors associated with referral of patients to the survivorship clinic (Table 3). In the discussion network, “betweenness,” an individual measure of network centrality, was the only significant correlate with patient referral. Betweenness measures a strategic position in a network capturing how often a person is in-between others by calculating the frequency each person is located on the shortest paths connecting all others in the network^16^. Participants with higher network betweenness, primarily physicians and social workers (labeled nodes, Fig. 1), were significantly more likely to have referred patients to the survivorship clinic (*p* = 0.025). Even while controlling for role at the clinic and network exposure, or the proportion of people in one’s personal network who had previously referred patients, betweenness centrality was still the most significant predictor of patient referral. None of the factors in the advice network had a significant association with patient referral.

**Table 3:** Multivariable logistic regression on patient referrals in discussion network.

	Estimate (SE)	*P*-value
Network exposure	−0.37 (0.98)	0.71
Betweenness centrality	0.43 (0.19)	0.025
Role at clinic^a^		
Physician Assistant/ Nurse Practitioner	−1.00 (1.30)	0.44
Physician	0.007 (0.76)	0.99
Scheduler/Other	−0.02 (0.76)	0.98
Social Worker	−1.44 (1.38)	0.30

*p < 0.05, **p < 0.01, ***p < 0.001.

^a^Nurses as Reference group.

## Discussion

In this study of social networks involving oncology clinicians, we found that social workers were prominent opinion leaders, that a strategic and influential network position (betweenness centrality) was associated with survivorship clinic referral, and that occupational role homophily was present. To our knowledge, social network analysis has not yet been utilized in the context of cancer survivorship. Our study provides preliminary data to identify opinion leaders and factors associated with survivorship clinic referrals, and to yield information toward future implementation of a network-based intervention to promote patient referral.

Homophily effects indicated that individuals were significantly more likely to have connections with others who have the same occupational role. Homophily effects have been demonstrated in past research across multiple health behaviors and outcomes^33–36^. Provider networks with more communication between all members are associated with higher quality of patient care^21^. The lack of an association for referral behavior as an attribute in the ERGMs suggests that referral is not spreading through the network indicating an unmet need for network interventions to promote this behavior. Greater communication could establish more connections to colleagues who refer patients to the survivorship clinic, increasing network exposures and facilitating providers to adopt this referral behavior. If information in the network is not flowing between people in different roles, referral behaviors may not spread between groups.

However, greater group homophily facilitates information flow and knowledge sharing amongst members and is more conducive to social influence^34,37^. Network homophily could prove advantageous at a cancer center, e.g., for interventions such as educational workshops or lecture series for multiple groups of clinicians and support staff that may, in turn, increase referral behavior (Supplementary Table 2). Interventions targeting specific occupational groups would create opportunities for colleagues from the same clinical role to interact with others who are referring patients, increasing awareness and promoting patient referral. More research is needed to determine whether a mixed group or single group intervention strategy is most effective.

Two social workers emerged as opinion leaders in the advice network. Since opinion leaders are effective agents of change in social networks^18–20,23,24^, social workers may represent an important yet overlooked group that can disseminate information and influence others to refer patients to specialized services. Engaging opinion leaders in the network to support referral of patients to the survivorship clinic could be an effective intervention. While opinion leaders will vary depending on the network surveyed, finding social workers as the key opinion leaders in our network highlights the importance of including individuals from a variety of clinical roles in addition to physicians in order to get a more complete picture of the network and to maximize intervention effectiveness.

While most respondents were aware of the existence of the survivorship clinic, only a minority actually referred patients. We observed that greater betweenness centrality in the discussion network significantly increased the odds of referring patients. In our case, a physician and two social workers had the highest betweenness centrality in the discussion network (labeled nodes Fig. 1). Betweenness is a measure that describes the strategic gatekeeper or mediator role someone has in a network^16^. They are distinct from opinion leaders, as they do not necessarily have the highest indegree. Individuals in the discussion network with higher betweenness centrality may interact with other network clusters, potentially allowing them to receive or spread information to different parts of the network more easily compared to people with lower betweenness. Based on their positions in the network, they may be more aware of certain aspects of patient care and encounter more clinicians who refer patients to the clinic. However, individuals with high betweenness will vary based on the social network being surveyed (i.e., we cannot generalize that physicians and social workers in all networks have higher betweenness).

In designing a network intervention, recruiting individuals with high betweenness centrality to disseminate information may be strategic for two reasons. First, they are already significantly more likely to have referred patients to the clinic, which means they have already adopted the behavior. Second, because of their network position, they have greater access to different clusters and may disseminate information more widely throughout the network. This finding could be applied to other clinical networks, since betweenness is a measure of network position, recruiting these members to implement an intervention would be advantageous.

Other institutions could apply these principles to promote survivorship clinic referral or adoption of potential interventions informed by our findings (see Supplementary Table 2). It should be emphasized that this study did not test the efficacy of these potential interventions, which would require a longitudinal study design with appropriate controls. However, even without a formal analysis, decision makers can benefit from understanding the social network framework. Often, presumptions about leadership are not necessarily aligned with the views of those embedded in the network, which is important to consider when changing practice norms. Surveys, informal conversations with staff, and communication with individuals in a variety of occupational roles can help identify network members in strategic positions that can influence change. Using the principles of social network analysis to promote change is an innovative and inclusive intervention approach that is applicable to both the academic and community-based practice setting.

This study has several strengths and some limitations. A significant strength is the use of social network analysis to understand survivorship clinic referral behaviors, an application not previously described, to our knowledge. Another strength is inclusion of varied clinical and clerical roles, which provides a “real world” look at how information is communicated in networks of a cancer center and indicates potential intervention strategies to improve referral. While the 42% response rate was somewhat lower than desired, it is within the range of 11–50% found in past physician survey studies, depending on speciality^38–40^. With regards to ERGMs, which model network ties, the proportion of missingness is larger and less ideal. However, network research has shown that the centrality measures we used, particularly indegree centrality (used to identify opinion leaders), are robust to missingness even with low network sampling levels^41,42^.

Another limitation is the missing demographic information for non-participants and incomplete survey data from select participants, which limited the sample size. Additionally, the cross-sectional nature of the study prohibits us from making causal inferences. There are other factors that may contribute to non-referral in addition to the ones posed here, but to reduce participant burden, we did not include survey questions about reasons for not referring patients to the clinic and did not attempt to delineate role-specific differences among respondents in regard to the referral process. Finally, inherent to social network analysis is that generalizability of specific findings may be limited because site-specific analyses are needed to account for the unique, contextual dynamics of social networks. Most survivors are treated in community-based cancer centers which may have a different communication dynamic compared with NCI-designated centers.

In conclusion, considering network structure and dynamics is a unique approach to designing interventions to improve patient referral and subsequently, quality of care. While most medicine is practiced in multidisciplinary teams, this study found that there is a strong effect of role homophily on communication networks, as providers still primarily interact with others who have the same clinical role. While professionally normative, this pattern may be slowing or limiting the spread of information throughout the network between occupations. Our study highlights the importance of having varied roles represented in a network intervention and provides promising evidence that using network analysis to identify opinion leaders and strategically important network members (high betweenness centrality) would be the first step for enhancing communication between team members, encouraging the spread of best practices and expediting new innovations in patient care.

## Supplementary information

Reporting Summary

Supplemental Material

## Data Availability

Raw datasets from this study can be accessed at https://github.com/sarahpiombo/SNA-clinicians.git^43^. Source data for Fig. 1 can be accessed as RDS files at https://github.com/sarahpiombo/SNA-clinicians.git^43^.

## Code availability

The R code used to conduct this analysis is available at: https://github.com/sarahpiombo/SNA-clinicians.git^43^.
