# Supplementary material for: Social networks of oncology clinicians as a means for increasing survivorship clinic referral
Source: Commun Med (Lond). 2022 Jul 15;2:89. doi: 10.1038/s43856-022-00153-0 (PMC9287406; doi:10.1038/s43856-022-00153-0)
Supplement: Supplementary file 1 — Reporting Summary [file 43856_2022_153_MOESM1_ESM.pdf]

nature research

Corresponding author(s): PIOMBO-COMMSMED-21-0526-T

Last updated by author(s): May 10, 2022

Reporting Summary

Nature Research wishes to improve the reproducibility of the work that we publish. This form provides structure for consistency and transparency in reporting. For further information on Nature Research policies, see our Editorial Policies and the Editorial Policy Checklist.

Statistics

For all statistical analyses, confirm that the following items are present in the figure legend, table legend, main text, or Methods section.

n/a	Confirmed	
☐	☒	The exact sample size (*n*) for each experimental group/condition, given as a discrete number and unit of measurement
☐	☒	A statement on whether measurements were taken from distinct samples or whether the same sample was measured repeatedly
☐	☒	The statistical test(s) used AND whether they are one- or two-sided<br>*Only common tests should be described solely by name; describe more complex techniques in the Methods section.*
☐	☒	A description of all covariates tested
☐	☒	A description of any assumptions or corrections, such as tests of normality and adjustment for multiple comparisons
☐	☒	A full description of the statistical parameters including central tendency (e.g. means) or other basic estimates (e.g. regression coefficient) AND variation (e.g. standard deviation) or associated estimates of uncertainty (e.g. confidence intervals)
☐	☒	For null hypothesis testing, the test statistic (e.g. *F*, *t*, *r*) with confidence intervals, effect sizes, degrees of freedom and *P* value noted<br>*Give P values as exact values whenever suitable.*
☒	☐	For Bayesian analysis, information on the choice of priors and Markov chain Monte Carlo settings
☒	☐	For hierarchical and complex designs, identification of the appropriate level for tests and full reporting of outcomes
☒	☐	Estimates of effect sizes (e.g. Cohen's *d*, Pearson's *r*), indicating how they were calculated

Our web collection on statistics for biologists contains articles on many of the points above.

Software and code

Policy information about availability of computer code

Data collection	Survey data was collected online through Qualtrics.
Data analysis	Data was processed and analyzed in R/RStudio:<br>R version 4.1.2 (2021-11-01)<br>Copyright (C) 2021 The R Foundation for Statistical Computing<br>RStudio 2021.09.0+351 "Ghost Orchid" Release (077589bcad3467ae79f318afe8641a1899a51606, 2021-09-20) for macOS

For manuscripts utilizing custom algorithms or software that are central to the research but not yet described in published literature, software must be made available to editors and reviewers. We strongly encourage code deposition in a community repository (e.g. GitHub). See the Nature Research guidelines for submitting code & software for further information.

Data

Policy information about availability of data

All manuscripts must include a data availability statement. This statement should provide the following information, where applicable:

- Accession codes, unique identifiers, or web links for publicly available datasets
- A list of figures that have associated raw data
- A description of any restrictions on data availability

The updated R code used to conduct this analysis is available at: https://github.com/sarahpiombo/SNA-clinicians.git

Field-specific reporting

Please select the one below that is the best fit for your research. If you are not sure, read the appropriate sections before making your selection.

☐ Life sciences ☒ Behavioural & social sciences ☐ Ecological, evolutionary & environmental sciences

For a reference copy of the document with all sections, see nature.com/documents/nr-reporting-summary-flat.pdf

Behavioural & social sciences study design

All studies must disclose on these points even when the disclosure is negative.

Study description	The study comprised a cross-sectional survey
Research sample	The sample included clinicians and clinical support staff who could potentially refer patients to the cancer survivorship clinic at the Cancer Center. Eligible clinicians included treating physicians (medical, surgical, and radiation oncologists), physician assistants, nurse practitioners, oncology clinic nurses, nurse navigators, social workers, and genetic counselors actively practicing at the cancer institute. Eligible clinical support staff included schedulers, direct care partners, and clerical referral specialists. All clinicians and staff had regular, direct contact with patients who met cancer survivorship clinic referral guidelines and played a role in the cancer survivorship clinic referral process.
Sampling strategy	Overall, 163 eligible individuals were identified from medical staff lists, department rosters, and managers and invited to participate. Recruitment was purposeful to include a representative range of cancer- and modality-specific treatment teams, departments and disciplines.<br>Sample size for the exponential random graph models can range from medium to large networks, typical from dozens to hundreds of network members (for example, networks as small as classrooms or as large as organizations). Therefore, a mid-size network (N=69) was sufficient to conduct analyses.
Data collection	Potential participants were invited by email to complete a confidential online survey through Qualtrics about professional social networks and patient referrals.
Timing	Data collection began in June 2018 and continued through the end of August 2018.
Data exclusions	Data was excluded if a participant began the survey but did not complete the survey. A survey was considered incomplete if a participant did not provide demographic information (i.e. occupation at clinic) or did not complete the social network component. For example, if a participant clicked the survey link and then quit, they were excluded from the analysis. 78 participants began the survey, but only 69 completed it. (N = 9 total participants excluded)
Non-participation	Response rate for the survey was 42%. The survey was sent to 163 eligible participants, and 69 completed the survey.
Randomization	There was no randomization or allocation of participants to groups.

Reporting for specific materials, systems and methods

We require information from authors about some types of materials, experimental systems and methods used in many studies. Here, indicate whether each material, system or method listed is relevant to your study. If you are not sure if a list item applies to your research, read the appropriate section before selecting a response.

Materials & experimental systems

n/a	Involved in the study	
☒	☐	Antibodies
☒	☐	Eukaryotic cell lines
☒	☐	Palaeontology and archaeology
☒	☐	Animals and other organisms
☐	☒	Human research participants
☒	☐	Clinical data
☒	☐	Dual use research of concern

Methods

n/a	Involved in the study	
☒	☐	ChIP-seq
☒	☐	Flow cytometry
☒	☐	MRI-based neuroimaging

Human research participants

Policy information about studies involving human research participants

Population characteristics	See above.

Recruitment	Potential participants were invited by email to complete a confidential online survey through Qualtrics about professional social networks and patient referrals.
Ethics oversight	University of Southern California Institutional Review Board

Note that full information on the approval of the study protocol must also be provided in the manuscript.
