# Supplementary material for: Social networks of oncology clinicians as a means for increasing survivorship clinic referral
Source: Commun Med (Lond). 2022 Jul 15;2:89. doi: 10.1038/s43856-022-00153-0 (PMC9287406; doi:10.1038/s43856-022-00153-0)
Supplement: Supplementary file 2 — Supplemental Material [file 43856_2022_153_MOESM2_ESM.pdf]

**Title:** Social Networks of Oncology Clinicians as a Means for Increasing Survivorship Clinic Referral

**Running Title:** Social Networks and Survivorship Clinic Referral

**Authors:** Sarah E. Piombo, MPH[1], Kimberly A. Miller, PhD[1,2], David R. Freyer, DO, MS[1,3,4,5], Joel E. Milam, PhD[6], Anamara Ritt-Olson, PhD[1], Gino K. In, MD[2,3,4] Thomas W. Valente, PhD[1]

[1]Department of Population and Public Health Sciences, Keck School of Medicine of the University of Southern California, Los Angeles, CA, United States

[2]Department of Dermatology, Keck School of Medicine of the University of Southern California, Los Angeles, CA, United States

[3]Department of Medicine, Keck School of Medicine of the University of Southern California, Los Angeles, CA, United States

[4]USC Norris Comprehensive Cancer Center, Los Angeles, CA, United States

[5]Cancer and Blood Disease Institute, Children's Hospital Los Angeles, Los Angeles, CA, United States

[6]Department of Epidemiology, School of Population Health, University of California Irvine, Irvine, CA, United States

**Corresponding author:** Sarah E. Piombo, MPH, Department of Population and Public Health Sciences, Keck School of Medicine of the University of Southern California, 1845 N. Soto St. Los Angeles, CA, United States 90032

Phone: (925)-595-7638 Email: piombo@usc.edu

**Supplementary Material**

Supplementary Table 1. Glossary of Social Network Analysis Terms[16]

| Term | Definition |
|---|---|
| Social network | Connections among people, organizations, political entities, and/or other units. |
| Social network analysis | A theoretical perspective and set of techniques used to understand these relationships and how they affect behaviors. |
| Indegree | The total number of nominations/ties an individual receives from others in the network. |
| Opinion leader | People in the network who have the highest indegree. Opinion leaders are in an advantaged position to access information about what is happening in the network. |
| Homophily | The tendency for people to affiliate and associate with others like themselves. |
| Betweenness Centrality | The frequency a person lies on the shortest path connecting everyone else in the network. Measures to what degree a node occupies a strategic position in a network. |

Supplementary Table 2. Potential Intervention Strategies Informed by Social Network Characteristics

| Network Characteristic | Intervention Strategy | Network Members |
|---|---|---|
| Opinion Leaders (Advice Network) | • Recruit opinion leaders (individuals with the highest indegree) to use their social influence in conversations with colleagues, educational outreach, or seminars to disseminate information about the benefits of referring patients to the survivorship clinic.<br>• Ensure that opinion leaders are already referring patients to the clinic. | Individuals with the highest indegree. In our network, these were the two social workers and one oncologist with the highest number of nominations. Opinion leaders will vary by network. |
| Betweenness Centrality (Discussion Network) | • Recruit discussion network members with high betweenness centrality to engage colleagues from different roles in conversations about the survivorship clinic.<br>• Utilize their network position to spread information to clusters that are difficult to reach. | Individuals with the highest betweenness centrality, calculated using social network analysis. In our network, these were primarily physicians and social workers occupying mediator or gatekeeper positions. These individuals vary by network. |
| Homophily | Option 1:<br>• Increase communication between individuals in different clinical and professional roles.<br>• Encourage collaboration and interdisciplinary team building.<br>• Focus on creating new professional connections to further increase exposure to others who are referring patients to the survivorship clinic. | Include physicians, nurses, physician assistants, social workers, and individuals from other clinical and clerical roles in the same event/intervention. |
| | Option 2:<br>• Utilize preexisting network homophily by hosting educational events, workshops, or lecture series specifically for physicians.<br>• Create opportunities for individuals from the same role to be exposed to others who are referring patients.<br>• Strong network homophily may result in individuals being more susceptible to social influence from others who have the same clinical role. | Design events/interventions that are segmented to only include individuals from the same role. For example, a seminar for oncologists on survivorship clinic referral, an educational event for social workers promoting awareness about follow-up care services, etc. |

**Supplementary Note 1: Network Survey**

We are interested in collecting information about referrals to the ____ Cancer Survivorship Clinic at the ____________ Cancer Center. This research uses social network analysis in which we ask you to identify yourself and your peers. These names are converted to random numbers and no identifying information will ever be shared with anyone. **All responses to these questions will be kept strictly confidential.** You may skip any question that you do not wish to answer or that make you feel uncomfortable. If you would like more information about this pilot study, please contact either _______ or ______. Ph: ____.

Q1. What is your first and last name? (Type the first few letters of your first name and a list will appear. If your name does not appear in the list simply type it in.)

Q2. What is your role at ____?

- ☐ Physician
- ☐ Physician Assistant
- ☐ Nurse Practitioner
- ☐ Clinic Nurse
- ☐ Nurse Navigator
- ☐ Social Worker
- ☐ Other_________________________

The ____ Survivorship Clinic at the _______ Cancer Center was started in 2017. It provides one-time assessments of cancer survivors who have completed treatment with the goal of improving long-term health after cancer treatment. Criteria to be seen at the ____ Clinic include:

- Treated when <50 yrs old,
- Treated with curative intent, and
- Treated using multimodal cancer therapy with potential for long-term toxicity

Q3. Prior to this survey, were you aware this clinic existed?

- ☐ Yes
- ☐ No
- ☐ Not sure/Don’t know

Q4. Please estimate how many patients you have seen in the prior 12 months who completed their treatment and met these criteria:

_________________

Q5. Have you referred any patients to the ____ Cancer Survivorship Clinic at the _______ Cancer Center?

- ☐ Yes
- ☐ No
- ☐ Not sure/Don't know

(IF YES) Q5a. Approximately how many patients have you referred to the ____ Clinic in the prior 12 months?

_________________

Q6. Please name up to 7 individuals at ______ (first and last name) with whom you **discuss any aspect of patient care** for all of your cancer patients. These individuals can be from any professional discipline or occupation, not necessarily one's own. Type the first few letters of the first name and a list will appear. If the name does not appear in the list simply type it in.

| First name | Last name |
|---|---|
| | |
| | |
| | |
| | |
| | |
| | |
| | |

Q7. Please name up to 7 individuals at _____ (first and last name) that you **go to for advice** for any aspect of patient care for all of your cancer patients. These individuals can be from any professional discipline or occupation, not necessarily one's own. Type the first few letters of the first name and a list will appear. If the name does not appear in the list simply type it in.

| First name | Last name |
|---|---|
| | |
| | |
| | |
| | |
| | |
| | |
| | |
